# Phenoseasonal subcanopy light dynamics and the effects of light on the physiological ecology of a common understory shrub, *Lindera benzoin*

**DOI:** 10.1371/journal.pone.0185894

**Published:** 2017-10-12

**Authors:** Janice E. Hudson, Delphis F. Levia, Sean A. Hudson, Harsh P. Bais, David R. Legates

**Affiliations:** 1 Department of Geography, University of Delaware, Newark, Delaware, United States of America; 2 Department of Plant and Soil Sciences, University of Delaware, Newark, Delaware, United States of America; 3 Department of Applied Economics and Statistics, University of Delaware, Newark, Delaware, United States of America; National Research Council of Italy, ITALY

## Abstract

The purpose of this work was to quantify the variation of subcanopy spatiotemporal light dynamics over the course of a year and to link it to the physiological ecology of the understory shrub, *Lindera benzoin* L. Blume (northern spicebush). Covering all seven phenoseasons of a deciduous forest, this work utilized a line quantum sensor to measure the variation in subcanopy light levels under all sky conditions at different times of the day. A total of 4,592 individual subcanopy measurements of photosynthetic photon flux density (PPFD, μmol m^-2^ s^-1^) were taken as 15-second spatially-integrated one-meter linear averages to better understand the dynamism of light exposure to *L*. *benzoin*. Both open (*n* = 2, one continuous and one instantaneous) and subcanopy location (*n* = 25) measurements of PPFD were taken on each sampling date in and near the forested plot (Maryland, USA). In addition, we explored the effect of four photointensity-photoperiod combinations on the growth of *L*. *benzoin* under controlled conditions to compare to field conditions. On average, understory PPFD was less than 2% of open PPFD during the leafed months and an average of 38.8% of open PPFD during leafless winter months, indicating that: (1) often overlooked woody surfaces intercept large amounts of light; and (2) spicebush within the plot receive limited light even in early spring before canopy leaf-out. Statistical results suggested phenoseason accounted for nearly three-quarters of the variation in incident radiation between the three plant canopy heights. Spicebush under controlled conditions exhibited the highest fitness levels at an intensity of 164.5 μmol m^-2^ s^-1^ for 12-hour duration. Similarly, spicebush growth in the field occurred at subcanopy locations receiving higher incidence of PPFD (*i*.*e*., >128 μmol m^-2^ s^-1^). Results suggest that the ecological niche for these plants is very specific in terms of light intensity.

## Introduction

Differences in insolation have a profound effect on the ecosystem ecology of a location, and light interception is a critical process that directly affects the physiological ecology of forest ecosystems [1–3]. It has long been known that exposure to light will affect the asymmetry of tree crowns [4] and the morphology of leaves on a single tree [5]. Leaves in the lower canopy of deciduous trees have thinner layers of spongy mesophyll and a lower number of chloroplasts than those in the upper canopy that intercept more light [5]. Thus, individual trees optimize the gain of photosynthate by having thicker leaves on the upper canopy and divest in the lower canopy with lower levels of light. Many of the phenological changes in trees are dependent on the changing spectral composition and quantity of solar radiation reaching the plants throughout the year. By combining the timing of the primary seasonal solar conditions in the northern hemisphere with that of canopy phenophases, Hutchison and Matt [2]defined the seven distinct phenoseasons for a temperate deciduous forest which this study utilizes. Those phenoseasons, from full leaf-off conditions, are winter leafless (November to February), spring leafless (February to April), spring leafing (April to May), summer leafing (May to June), summer fully-leafed (June to August), autumnal fully-leafed (August to October), and autumnal partially-leafed (October to November). Relatively few studies (*e*.*g*. less than 10) have utilized phenoseasons as a meaningful construct with which to observe and measure changes within a forest.

The dynamics of understory light are greatly impacted as a forest undergoes the phenological changes associated with these phenoseasons. Understory light availability, including photosynthetically active radiation (PAR, 400–700 nm), has been shown to vary at all spatial and temporal scales [2,6–10]. Previous studies of PAR have employed individual spot quantum sensors [11], light sensing diodes [12], spherical fiber optic cable sensors [9], solarimeters [2], spectroradiometers [13] and chemical light meters [14]. Sensors have typically been placed at multiple depths along the canopy-subcanopy gradient, usually at less than three locations [2,13]. Sensors might be turned on for only a few days during a season or for moments of each cloud condition for growing seasons only [4,6,7, and others]. In a few studies, readings were taken for only one day in each season. While some studies have taken millions of PAR readings in a field site during a growing season [15], the readings are often stationary and constrained by sensor diameter and therefore limited spatially [13]or, when using a larger diameter or fish-eye sensor, precision is compromised [16]. Published works of such large data sets often are only inclusive of particular days. Subcanopy photosynthetic photon flux density (PPFD), a measure of PAR, also varies with canopy species and stand density, ranging from 31% of total light in slash pine stands [12]to as little as 1.4% of total light in a hardwood stand [14]. The variability of subcanopy light energy, in concert with other limiting factors in the ecosystem, may alter the species composition, abundance, and fitness of understory plants in a given ecosystem. Competition in the understory is fierce; plants in shade environments compete to utilize solar energy and available nutrients for growth and reproduction, while nutrient and radiative inputs are constantly changed by canopy species [6,15,17,18]. Acquisition and allocation of resources, and efficient use of those resources, is of the utmost importance in the survival of understory plants.

It is well known that solar radiation, and PAR, decrease exponentially as a function of LAI [19]. In deciduous forests, with a canopy leaf area index (LAI, m^2^ m^-2^) commonly ranging from 4 to 6 m^2^ m^-2^ during the foliated phenoseasons, light exposure is critical to understory plants which need to maximize carbon gain before the canopy leaves emerge as well as during short-lived sunflecks [20]. Although photosynthetic response varies greatly among plants, shade-tolerant plants have adapted physically and chemically to thrive in highly variable light environments. They often require less light energy or fewer hours of daylight to complete biological processes [19]. They typically have broader, thinner leaves than shade intolerant species, grow more laterally, and are generally more efficient in their use of intercepted photons than light adapted plants [19].

One such plant, *Lindera benzoin* L. Blume (northern spicebush), is common and ecologically significant in the understory of mid-Atlantic deciduous forests. *Lindera benzoin* is the dominant understory species within our plot and is often found under *L*. *tulipifera*. The spicebush’s drupes are an important food source for Passerine and Phasianidae, especially during fall migration. Leaves are a food source for white-tailed deer and other herbivorous mammals, and an invaluable habitat for small to medium sized mammals, songbirds, pheasants, and insects—specifically, the namesake spicebush swallowtail and spicebush silkmoth. Many physiological traits of *L*. *benzoin*, including growth, herbivory defense, and reproduction, have been shown to be greatly affected by light intensities [21–24]. While inhibited by light conditions greater than 25% of open PPFD and less than 1% open PPFD [25], stem growth and flower and fruit production by *L*. *benzoin* plants of all ages is greater in sun patches and along forest edges [22]. In a study observing plasticity in response to light changes, *L*. *benzoin* demonstrated greater amounts of new stem growth, lower stomatal density, higher photosynthetic rate, and lower leaf thickness at light levels between 191 and 391 μmol m^-2^ s^-1^ in just 11 weeks [25]. Leaves of spicebush grown in sun conditions are often tougher, have lower water content, and typically experience less herbivory than leaves of spicebush grown in shade conditions [21].

Success in the field impacts a plant’s ability to nurture insects that depend on them. In the case of *L*. *benzoin*, populations of spicebush swallowtail butterfly and spicebush silkmoth are both affected by the health and nutritional status of these plants, as they rely on them almost exclusively in order to survive even the first instar stage [26]. This raises a question as to how variable light exposure is to the understory plant *L*. *benzoin*, and how these cumulative differences in light exposure alter the leaf biochemistry over time, especially when healthy plants are critically needed for other native organisms such as races of *papilionidae* and *saturniidae*. How light exposure to *L*. *benzoin* changes over the course of a year is still not well understood. Answers to such questions in both the field and laboratory are necessary to a gain a holistic and more comprehensive understanding of the physiological ecology of *L*. *benzoin* in the understory.

The aims of this study were to quantify PAR in the subcanopy and its spatial and temporal variation within and among phenoseasons in a mid-Atlantic deciduous forest, as well as to gain a better understanding of how this PAR variability affects woody understory shrubs, specifically *L*. *benzoin*. This work is novel because it combines (1) measurements of PPFD (*n* = 4,592) using a line quantum sensor in the open and at 25 locations and three elevations below the canopy across all phenoseasons with (2) a suite of laboratory experiments that examine *L*. *benzoin* response to varying photointensity- photoperiod conditions. To our knowledge, no other study has collected PAR measurements as often and at as many locations and heights, for all seven phenoseasons. This work builds upon previous studies of subcanopy radiation regimes, and contributes to gaps in the literature pertaining to PAR variability, specifically as it relates to the often under-utilized phenoseason construct, the effect of PAR availability on the physiological ecology of an ecologically significant understory plant, and measurement of subcanopy light dynamics at the microscale [27]. Additionally, while PAR can be model-derived using remotely sensed data, the contributions of large, *in-situ*, subcanopy datasets to future analyses cannot be underestimated.

## Materials and methods

### PAR measurement in the field

**Fig 1:**
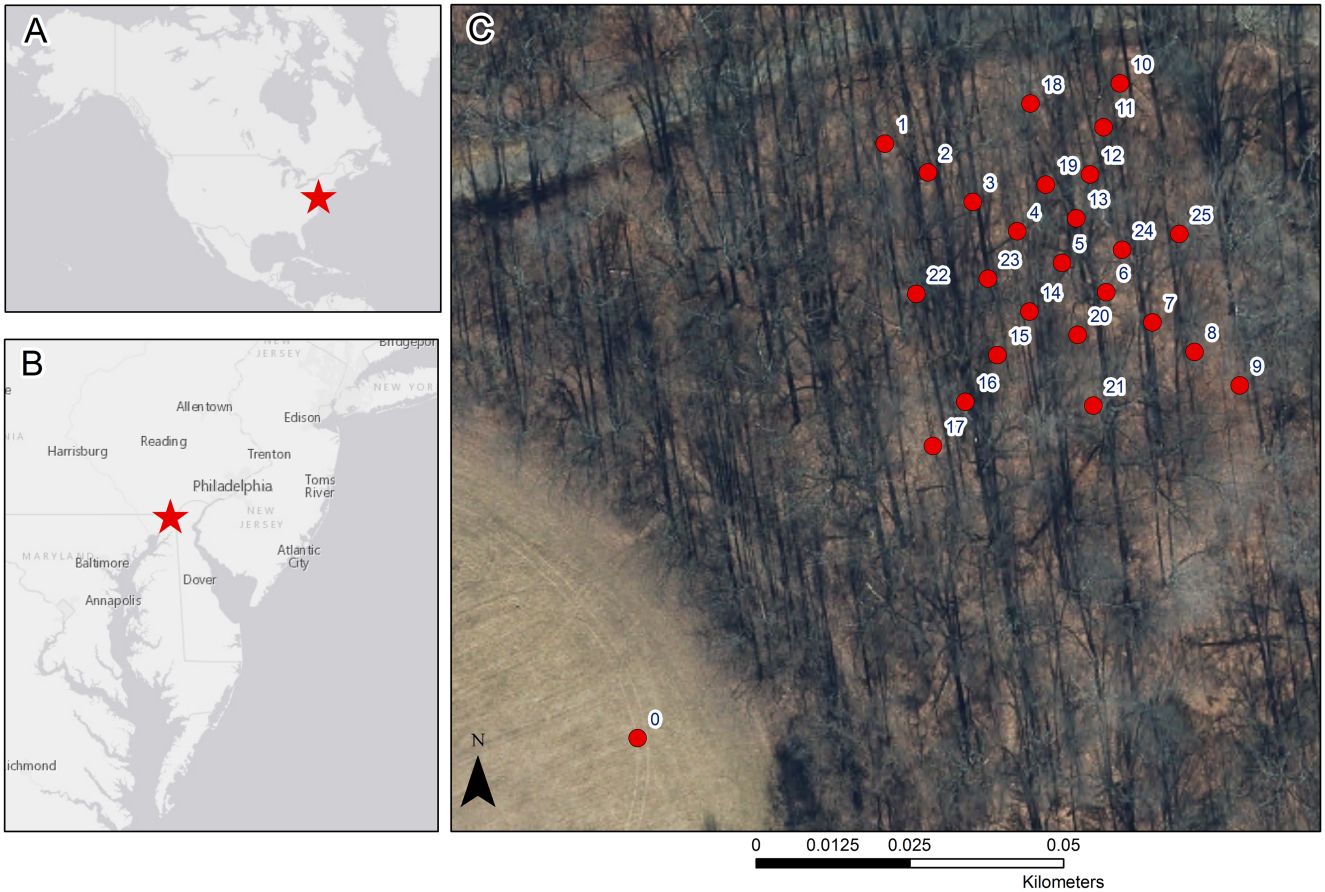
Map of the study area. The study area in relation to (A) the United States, (B) the mid-Atlantic region, and (C) the arrangement of the field site, where PPFD measurements were taken at 25 understory locations (1–25) and the open location (0). At locations 5, 8, 14, 15, and 23, readings were taken within and below *L*. *benzoin* canopy.

Field work was conducted in an expanded 1800 m^2^ research plot in Fair Hill Natural Resource Management Area (NRMA) in Cecil County, MD, USA (Fig 1B; 39°42'37.0"N 75°50'56.0"W). The NRMA is managed by the state of Maryland Department of Natural Resources with whom the university has a long-term use agreement. This field study did not involve endangered or protected species. This region lies within the humid subtropical (Cfa) climate classification of the Köppen system, receiving 1205.2 mm annual mean precipitation, which falls almost evenly throughout the year [28]. The 30-year mean maximum temperature is 19.1°C and the 30-year minimum temperature is 6.7°C [28]. Similar to other deciduous forests in the mid-Atlantic, dominant tree species in the research plot are *Fagus grandifolia* Ehrh. (American beech) and *Liriodendron tulipifera* L. (yellow poplar), with few *Acer rubrum* L. (red maple), *Carya* spp. (hickory), and *Quercus* spp. (oak). Average tree age within the stand is approximately 55 years. The plot contains 42 trees having a diameter at breast height (DBH) greater than 10 cm, and has a stand density of 233 trees/ha. The stand mean DBH is 39.5 ± 28.8 cm. Based on a plot inventory conducted in October of 2014, canopy heights range from 5.5 m to 30 m, with a mean height of 23 m. The stand basal area is 43.7 m^2^ ha^-1^. The plot has a woody area index of 1.21 ± 0.01 m^2^ m^-2^ and a leaf area index of 5.12 ± 0.17 m^2^ m^-2^. Soils in the area are primarily loam and silt loams of igneous, metamorphic, phyllite and schist parent material [29]. Within the plot, slopes range from 0 to 30% [29] with a primarily northeasterly aspect. Each of these plot characteristics describes the amount of surface within the plot that has the ability to intercept light and thus influence the amount of light reaching the forest floor.

PPFD was measured using a LI-COR LI-191 Line Quantum Sensor and LI-COR LI-250A light meter (LI-COR Lincoln, NE, USA), which can take an instantaneous measurement, or of recording a fifteen-second spatially-integrated average of the PPFD in units of μmol m^-2^ s^-1^, along the length of a one-meter quartz rod conductor under an acrylic diffuser (for full specifications, see the LI-COR website, www.licor.com). The one-meter, fifteen-second average integration of the LI-191 has a few advantages over spot meters for this study, including size (spot sensors are often smaller than a dime), lack of need for cosine correction (because the sensor is located under an acrylic diffuser), and most importantly, each measurement captures a much more representative sample of the overall field of PPFD (due to a larger sensor area and because averaged readings are not as susceptible to the inherently high spatial and temporal variability of subcanopy PPFD).

Subcanopy PPFD measurements were acquired non-simultaneously at a height of 1.75 m, using an instrument-attached circular spirit level, at twenty-five marked locations within the plot (Fig 1C, locations 1–25). An additional location in an open field ~100 m from the plot allowed for an open PPFD reading to compare with subcanopy measurements (Fig 1C, point 0), as well as with values from an additional quantum sensor installed at the nearby (~1 km) meteorological station managed by the Delaware Environmental Observing System (DEOS, not shown on map). Measurements were collected at each of the 25 subcanopy locations two to three times a week for one calendar year with exclusions for days with active precipitation and for site access restrictions due to weather and other closures of the property. Measurements began at the end of November 2013 (after full leaf-off conditions) and ended mid-November of 2014 (*n* = 4,592). We set up the field site to collect measurements in the most timely manner to reduce changes in light conditions during collection. Most locations within the plot have a litter layer with no understory vegetation except for short-lived, seasonal flowers in the spring, and few locations have growth of ferns or jack-in-the-pulpit. Thus, it was easy to discern by sight areas in the understory where spicebush were growing densely. To better understand light availability at these locations, we framed our methodology in a way that would allow us to measure the light above, within, and under the spicebush canopy. At the five subcanopy locations within the plot where there was dense growth of *L*. *benzoin* (Fig 1C, locations 5, 8, 14, 15, and 23), PPFD measurements were conducted above the *L*. *benzoin* canopy (at a height of 1.75 m), within the *L*. *benzoin* canopy (0.75 m), and beneath the *L*. *benzoin* canopy (0 m) on each visit.

**Fig 2:**
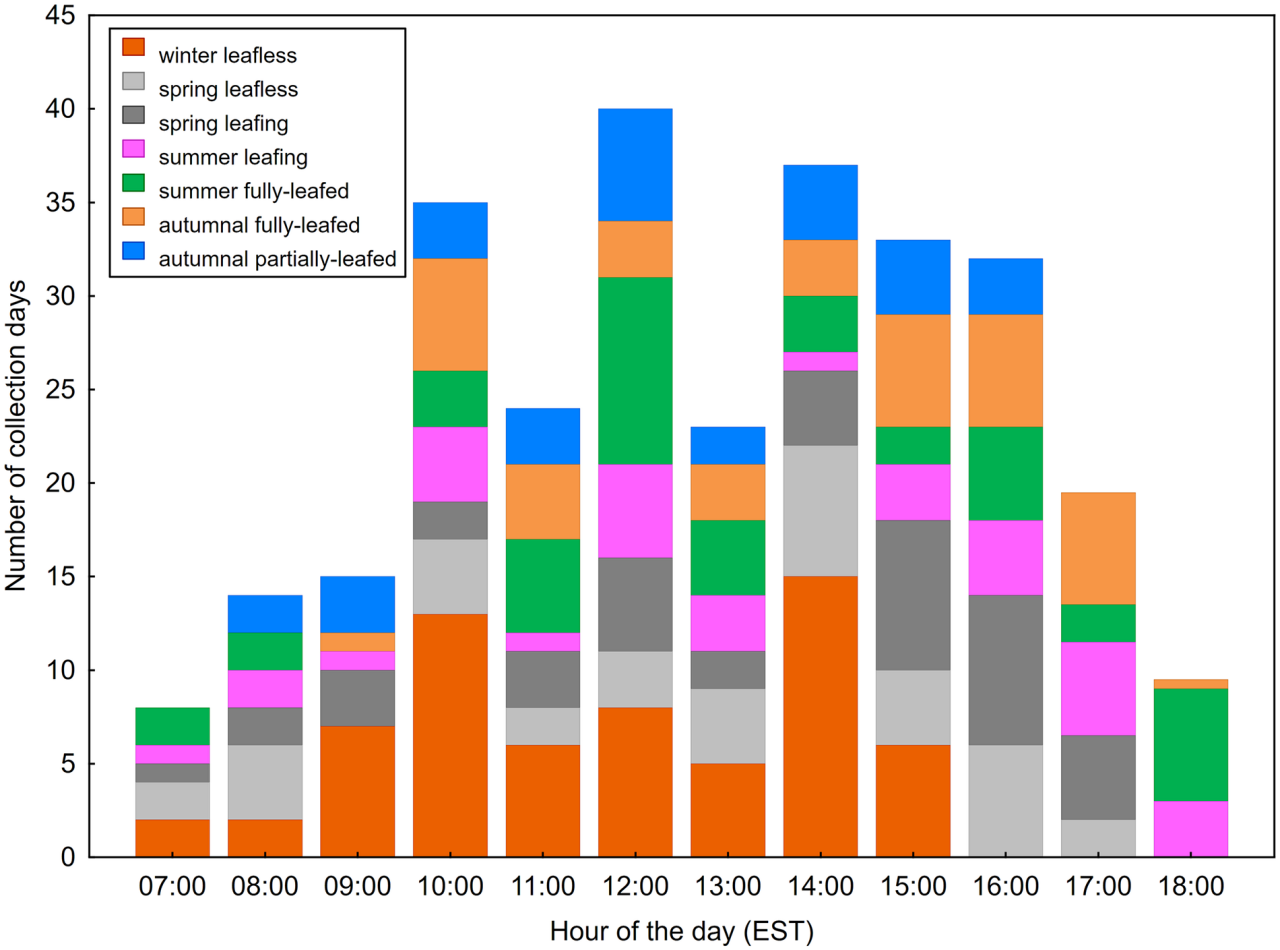
Collection days by hour and phenoseason. Stacked histogram showing, for a given hour, the number of days on which observations were made, regardless of sky condition (except days with active precipitation). Individual phenoseasons are color-coded, and stacks represent the total number of collection days during that daylight hour. The figure makes visible a clear pattern of seasons; winter leafless and spring leafless phenoseasons are short, and sunset occurs early (around 16:00 EST), while the fully-leafed phenoseasons are long with longer days.

The week, year, month, day, Julian date, phenoseason, visual sky condition, and PPFD measurements and associated times were recorded using a field sheet created this study, before being entered into a database. Visual sky conditions for each field visit were described in Oktas, a meteorological description of cloud cover in 1/8th increments of sky, where 0 is clear sky, 4 is 50% sky cloudiness, and 8 is completely overcast. To capture the broadest range of light and cloud conditions possible, trips to the field site were tracked using a spreadsheet in which the number of visits during a 15-minute interval for each phenoseason could be recorded. In this way, measurements could be collected during most daylight hours regardless of sky condition, except for instances of active precipitation. For ease of understanding, the number of collection days completed during a phenoseason’s daylight hours has been condensed into a one-hour interval in Fig 2. Because measurements were made on foot, every attempt was made to complete the entire set of measurements in the field within a half-hour period to avoid substantial shifts in light.

During statistical analysis of the field measurements, no outlier values were removed from the dataset because we are confident that outlier values here represent important, and often the most interesting aspects of the data. In this setting, such values represent sunflecks and spatiotemporal hotspots which can occur when radiation penetrates persistent canopy gaps for a longer duration than more highly variable sunflecks.

### *Lindera benzoin* treatments in the growth chamber

To quantify spicebush adaptation to simulated light regime conditions rather than seasonality, a growth chamber was arranged for two simultaneous treatments in each of two temporally separate experiments. The first experiment was intended to simulate an exaggerated version of full-sun PAR conditions, while the second was meant to simulate more realistic subcanopy or shade conditions. Both experiments were conducted using the same set-up and temperature and humidity settings, so that the light conditions would be the only variable. For each experiment respectively, *L*. *benzoin* seeds purchased from Horizon Herbs (Williams, OR, USA) were germinated in divided containers in PRO-MIX^®^ (Premier Tech, Quebec, Canada) and grown in natural light at room temperature in the laboratory for approximately 60 days. After this time, plants were re-potted in fresh PRO-MIX^®^ in individual pots and placed into the base of a Conviron PGC20 Reach-In Plant Growth Chamber (Winnipeg, Manitoba, Canada) programmed at a constant 24°C and 68% relative humidity for 35 days for both experiments. At the time of use, the growth chamber was equipped with 400W metal halide light bulbs (MH400/U//ED28, Philips Lighting North America).

To maximize resources, each of the two treatments in the experiments were set up to receive a different photointensity for a standard photoperiod by elevating one treatment 55 cm above the other, thereby increasing the PPFD for the elevated plants. For Experiment 1, intended to simulate high-intensity and long photoperiod, the overall photointensity within the growth chamber was set to 350 μmol m^-2^s^-1^. This value was selected based on our experience with maximum PPFD values in the subcanopy at our site, as well as a reasonable mid-point based on field work of Niesenbaum and Kluger [30]who found that in a similar deciduous forest containing spicebush in Northampton County, PA, the average PAR for shade and sun environments during the month of July were 48.01 μmol m^-2^s^-1^ and 658.3 μmol m^-2^s^-1^ respectively. With the growth chamber programmed to 350 μmol m^-2^s^-1^ overall intensity, PPFD was manually measured at the soil surface to be 517.9 μmol m^-2^s^-1^ for the elevated upper treatment and 366 μmol m^-2^s^-1^ for the lower treatment using an LI-190 Quantum Sensor in conjunction with a LI-COR LI-250A light meter (LI-COR, Lincoln, NE, USA). The program was set to run 16 hours of light and 8 hours of darkness for Experiment 1. Overall photointensity for the second experiment was set to 150 μmol m^-2^s^-1^ and was intended to simulate a lower intensity, shorter photoperiod (subcanopy or shade) environment. At this intensity, the upper and lower treatments of Experiment 2 were recorded at the soil surface to be 164.5 μmol m^-2^s^-1^ and 118.06 μmol m^-2^s^-1^, respectively, for 12 hours of light and 12 hours of darkness. These values were much closer to light conditions in our field site, where we knew that average PPFD at the center of the plot just above spicebush growth was 125.79 μmol m^-2^s^-1^. The growth chamber was unable to be set for a lower photointensity than 150 μmol m^-2^s^-1^, thus limiting the lower range of PPFD for this aspect of the study.

Using a Model SC-1 Leaf Porometer (Decagon Devices, Inc., Pullman, WA, USA), stomatal conductance measurements were taken on all intact leaves twice for each experiment; once during the second-to-last week in the growth chamber, and again one week later, during the last week in the growth chamber. Living growth chamber plants were non-destructively measured for leaf area, number of nodes, inter-nodal distances, and total node height. At the end of the 35-day growing stage, plants were harvested for destructive measurements including weight and dry weight of roots and shoots.

### Statistical analysis

We employed statistical methods in STATISTICA 13.2 to determine differences in the PAR variations. These methods include comparing descriptive statistics and *t*-tests among the central tendencies and dispersion of the variables. Analysis of Variance (ANOVA) was used to evaluate PAR variation in the shrub canopy. A Tukey-Kramer Honest Significant Difference test was employed to ascertain differences between plants in the growth chamber experiments. The Tukey-Kramer Honest Significant Difference test (or Tukey's Range test) is designed to identify means which differ significantly by comparing all pairs of means and utilizing a studentized (i.e., *t*-values) distribution [31]. It assumes independent observations, the groups are normally-distributed, and homogeneity of variance (that is, within-group variance is the same for all groups).

## Results

### PAR measurement in the field

#### PAR variation across phenoseasons

**Fig 3:**
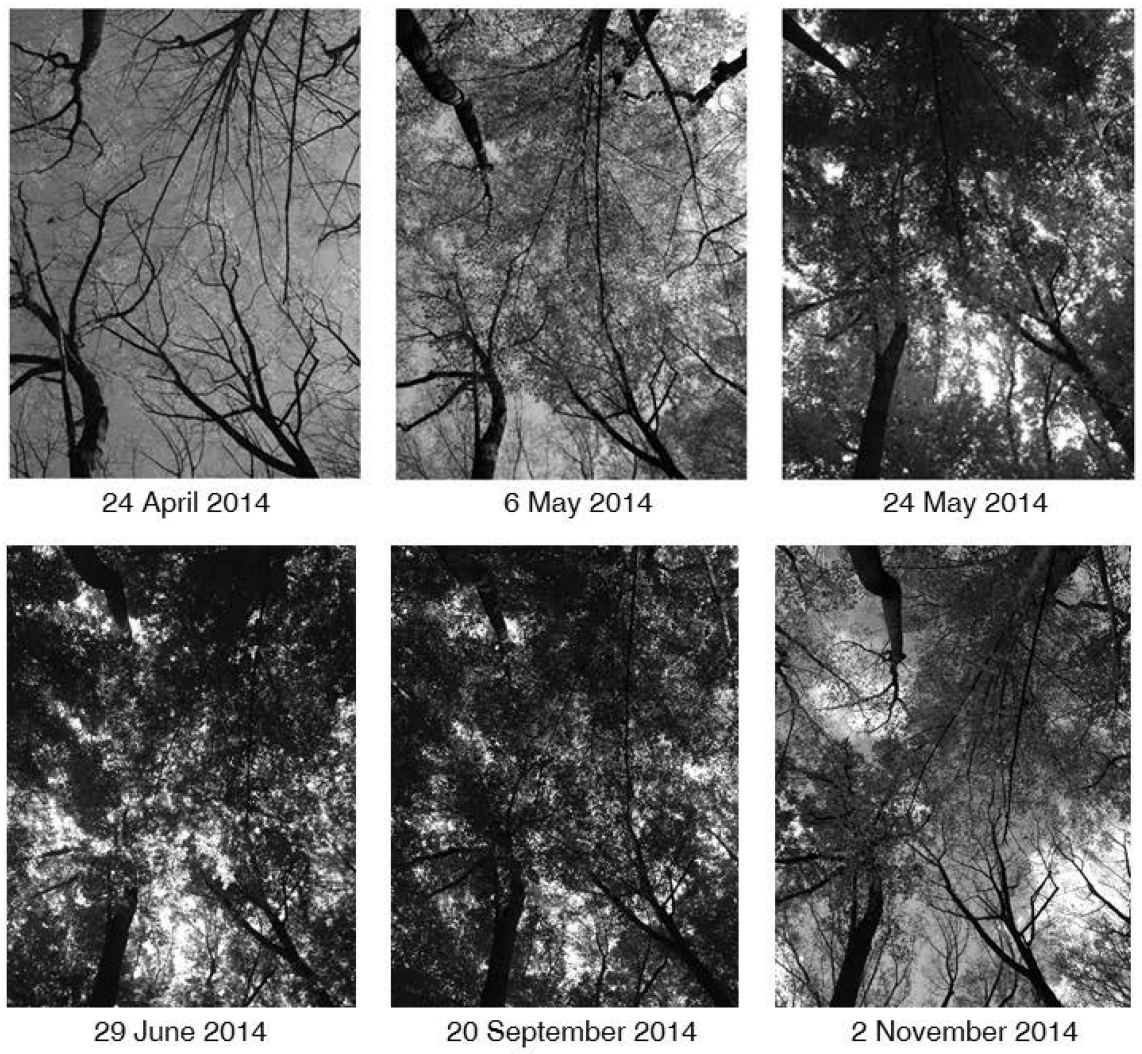
Vertical reference images in different phenoseasons. Vertical referential photographs of canopy cover at the same location throughout the course of the study, showing the progression of phenophases from bud burst (24 April; spring leafing), leaf out (6 May; spring leafing), intermediate and maturing canopy (24 May & 29 June; summer leafing), mature closed canopy (20 September; autumnal fully-leafed), and the beginning of abscission (2 November; autumnal partially-leafed) of 2014.

**Fig 4:**
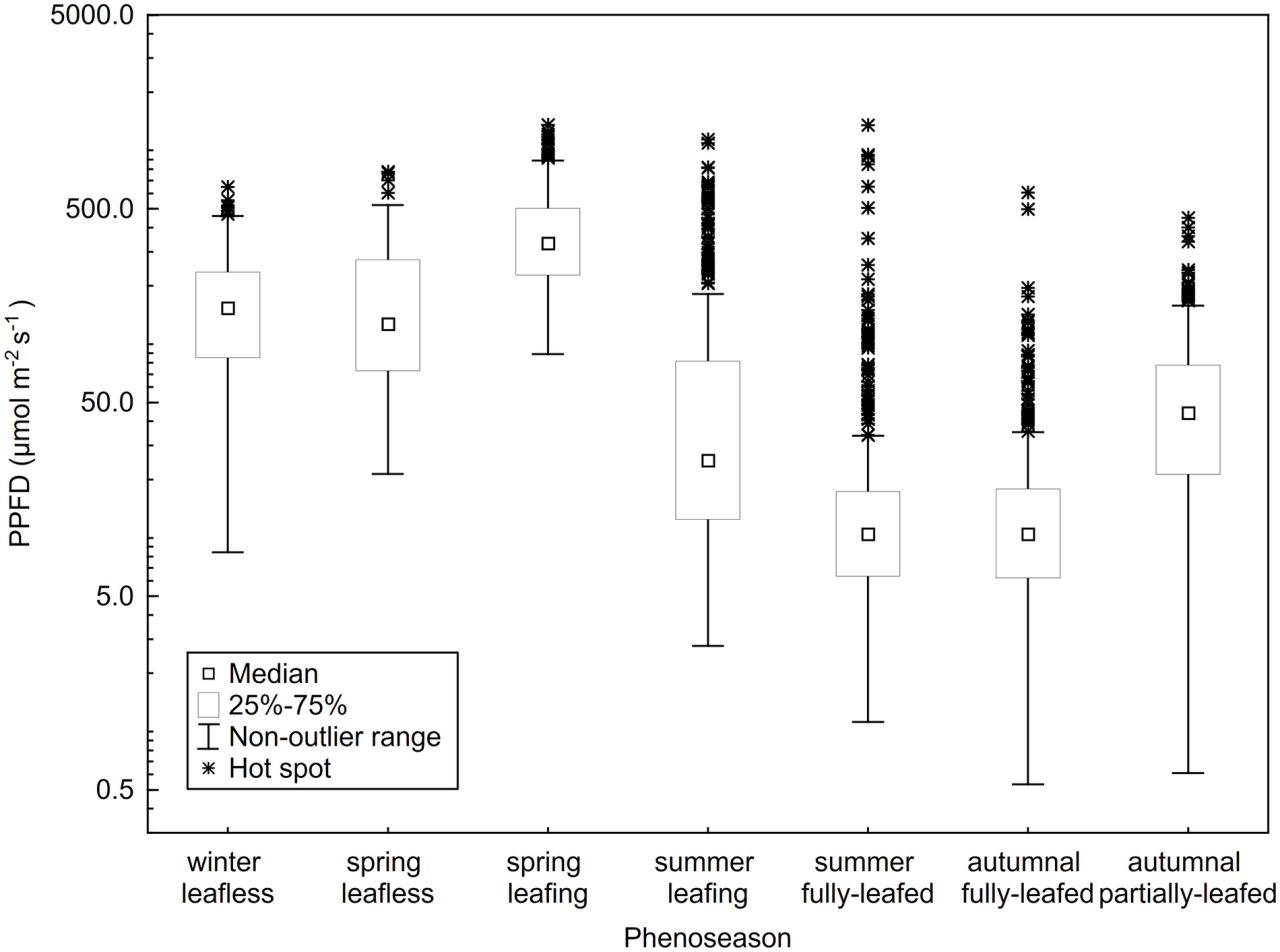
Range of PPFD values in different phenoseasons. Range of PPFD values for each phenoseason at the 25 subcanopy locations a height 1.75 m. Plot shows the trend of median subcanopy PPFD decreasing with increasing canopy closure, and the number of hot spots increasing during this same time period due to high-energy sunflecks through the closed canopy.

We compared measurements of PPFD from each location, from three canopy heights, and across seven phenoseasons. Vertical referential photographs in Fig 3 show the progression of leaf out, to canopy closure, and the beginning of leaf abscission through these seven phenoseason at the same location under the canopy. Each subcanopy location at 1.75 m height demonstrated a similar pattern (Fig 4): an increase in subcanopy flux from the winter leafless to the spring leafing phenoseason, a large decrease in the subcanopy flux as canopy leaf-out occurred accompanied by an increase in the number of extreme values of PPFD for the summer leafing phenoseason, a further decrease in subcanopy flux for summer and autumnal fully-leafed phenoseasons before a slight increase for the autumnal partially-leafed phenoseason (Fig 4). Although there was some variance in the total cumulative energy received between each of the 25 subcanopy locations, the phenoseasonal means were not significantly different, and each location generally followed this same pattern of seasonal flux and received near the same cumulative annual flux (126.95 ± 21.67 μmol m^-2^ s^-1^).

The spring leafless and early spring leafing phenoseasons had lower open PPFD values and higher, more variable subcanopy PPFD values than the summer phenoseasons. Late summer leafing and fully-leafed phenoseasons had PPFD values that were higher in the open, lower and less variable values in the subcanopy, with distinct hot-spots (or sunflecks) because of canopy closure, sky conditions, and the position of the sun.

Understory PPFD had, overall, a greater range of non-outlier values during the unfoliated phenoseasons, but “extreme-outlier” cases (hot spots and sunflecks) increased as median PPFD values and interquartile variability decreased with foliated phenoseasons. The summer fully-leafed and autumnal fully-leafed phenoseasons had the greatest instances of extreme values (measurements often went from near zero to many hundreds of μmol m^-2^ s^-1^). The spring leafing and summer leafing phenoseasons had the highest standard deviations, 259.48 and 218.76 μmol m^-2^ s^-1^, respectively.

**Fig 5:**
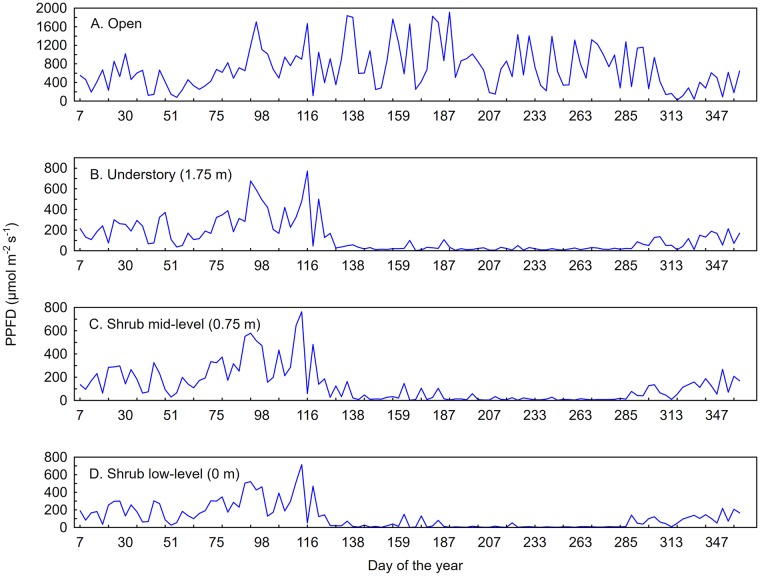
Mean annual PPFD by day of the year. Continuous line plot of mean annual PPFD for the entire study in the open (A), in the understory above the shrub canopy (B), within the shrub canopy (C), and below the shrub canopy (D). Canopy closure occurs near day 129 which causes a reduction in PPFD at all heights in the subcanopy, while PPFD in the open remains high and variable. PPFD in the subcanopy begins to increase again around day 285 as leaves abscise.

Annual PPFD was highest in the open location (Fig 5). The variance within the time series of daily open PPFD values was likely due to weather patterns and sky conditions, while the overall trend of open PPFD increased starting around Day 75 (16 March), and reached a maximum around Day 187 (06 July) before generally decreasing after Day 230 (18 September) due to earth-sun geometry. The understory, shrub mid-height, and shrub low-height PPFD series increased from Day 1–120 (30 April) and roughly followed that of the open, but at only an average of 50% of the incident flux (Fig 5). There was a rapid decrease around Day 129 during the summer leafing phenoseason, at which time subcanopy values dropped to less than 12% of open flux after canopy leaf-out until a slight increasing trend began around Day 285 during the autumnal partially-leafed phenoseason (Fig 5). Relative to the open PPFD location, the daily maximum peaks in all the subcanopy PPFD series were muted but, unexpectedly, the shrub mid-height often received more radiative energy than the above shrub-canopy readings (Fig 5). Annually, the understory, shrub mid-height, and shrub low-height PPFD values were 23 ± 20%, 36 ± 66%, and 33 ± 60% of the open values, respectively. For the foliated period only, those values dropped to 3 ± 2%, 5±7%, and 4 ± 8% of open PPFD values, respectively. For values of median flux as a percent of open PPFD for each height and phenoseason, in addition to other descriptive statistics that assist in the describing the variability and skewness of the dataset, please refer to Table 1.

**Table 1:** Phenoseasonal PPFD for each canopy height in field conditions.

Phenoseason	% partly cloudy days (> 4 Oktas)	% overcast days (8 Oktas)	Height of PPFD measurement (m)	Valid *n*	Mean (± 1 SD) (μmol s^-1^ m^-2^)	Median PPFD (μmol s^-1^ m^-2^)	Lower Quartile (μmol s^-1^ m^-2^)	Upper Quartile (μmol s^-1^ m^-2^)	Median PPFD as a % of Open PPFD	Minimum (μmol s^-1^ m^-2^)	Maximum (μmol s^-1^ m^-2^)
*winter leafless*	41.5	11.9	open	21	478.8 ± 245.8	504.2	281.1	617.3	-	42.9	1017.9
			1.75	667	181.2 ± 124.6	158.0	87.2	244.9	31.3	8.4	1017.9
			0.75	21	170.7 ± 79.4	169.7	112.6	231.8	33.7	55.1	298.6
			0	21	159.8 ± 78.5	167.0	102.8	208.5	33.1	37.5	299.8
*spring leafless*	65.1	43.6	open	13	346.7 ± 210.1	330.0	145.3	460.3	-	84.0	682.1
			1.75	399	178.7 ± 133.3	129.7	74.2	286.9	39.3	21.4	777.6
			0.75	14	190.1 ± 113.1	181.6	91.8	324.9	55.0	29.1	373.0
			0	14	180.9 ± 107.9	171.7	86.5	300.3	52.0	26.0	348.6
*spring leafing*	46.7	6.6	open	15	943.9 ± 364.2	904.8	683.4	1111.0	-	491.0	1704.7
			1.75	435	415.2 ± 259.5	341.7	229.7	520.4	37.8	88.7	1704.7
			0.75	15	374.1 ± 206.9	316.0	199.7	551.6	34.9	59.2	761.8
			0	15	338.3 ± 184.7	296.6	175.2	505.1	32.8	52.4	713.8
*summer leafing*	50	14.5	open	14	792.6 ± 536.5	747.6	350.0	1051.3	-	117.1	1839.0
			1.75	406	105.5 ± 218.8	26.5	12.9	98.3	3.5	2.8	1839.0
			0.75	14	92.3 ± 128.3	30.6	13.3	139.1	4.1	8.0	480.9
			0	14	68.3 ± 124.2	20.2	70.8	124.2	2.7	471.4	11.3
*summer fully-leafed*	45.5	16.9	open	22	916.4 ± 545.8	854.7	527.5	1269.0	-	146.6	1913.3
			1.75	637	56.8 ± 211.3	10.8	6.5	18.8	1.3	1.1	1913.3
			0.75	22	29.4 ± 39.4	13.3	5.4	32.2	1.6	1.6	147.0
			0	22	26.6 ± 42.0	7.9	3.8	18.7	0.9	1.1	151.5
*autumnal fully-leafed*	32.3	25.4	open	17	841.9 ± 431.9	741.1	495.1	1311.8	-	221.5	1428.0
			1.75	493	47.1 ± 174.1	11.1	6.3	19.3	1.5	0.5	1428.0
			0.75	17	9.5 ± 6.7	7.7	5.4	11.6	1.0	1.7	27.4
			0	17	6.3 ± 2.7	7.2	4.1	8.1	1.0	2.2	10.3
*autumnal partially-leafed*	58.4	20.9	open	14	536.8 ± 453.3	298.7	164.4	992.6	-	23.8	1278.8
			1.75	397	77.0 ± 133.0	44.9	22.5	85.1	15.0	0.6	1278.8
			0.75	13	57.5 ± 44.2	46.0	18.5	77.4	15.4	8.5	134.6
			0	13	56.5 ± 45.5	46.9	10.5	95.1	15.7	7.6	141.5

#### PAR variation by phenoseason

To highlight the variability between cloud conditions during similar radiative conditions in this large dataset, three days were chosen from each phenoseason when readings were collected as near to solar noon as possible, to represent one clear, one partly cloudy, and one overcast day (Table 2). For some phenoseasons, no solar noon-cloud condition combinations were available so the nearest condition was used. Table 2 highlights the increasing radiative energy (from partly cloudy conditions) with diffuse light of overcast conditions, both in the open and subcanopy during the winter and spring leafless months [19]. In contrast, overcast conditions during the summer tend to decrease the open PPFD, substantially reduce subcanopy mean PPFD, and because of diffuse lighting, reduce variability of subcanopy PPFD

**Table 2:** Summary characteristics of selected days in 2014.

Phenoseason	Sky Condition	Oktas	Day	Collection Start—End Time (EST)	Open PPFD (μmol m^-2^ s^-1^)	Subcanopy Mean ± 1SD PPFD (μmol m^-2^ s^-1^)
*winter leafless*	clear	0	30-Jan	12:21–12:51	1017.9	184.69 ± 30.15
	partly cloudy	4	16-Jan	13:16–13:44	416.1	225.62 ± 159.80
	overcast	7	4-Feb	11:55–12:23	606.1	295.21 ± 39.54
*spring leafless*	clear	0	28-Feb	*07:34–08:07	245.1	50.57 ± 17.03
	partly cloudy	5	10-Feb	*16:14–16:35	144.6	74.70 ± 11.08
	overcast	8	16-Mar	13:15–13:45	682.1	334.77 ± 27.78
*spring leafing*	clear	0	26-Apr	12:50–13:21	1666.9	772.28 ± 268.74
	partly cloudy	3	1-Apr	11:37–12:07	1196.4	675.98 ± 274.28
	overcast	8	23-Mar	12:08–12:30	716.5	312.34 ± 36.16
*summer leafing*	clear	1	26-May	15:09–15:38	1084.7	32.04 ± 28.98
	partly cloudy	5	3-May	12:14–12:46	1051.3	499.56 ± 248.24
	overcast	8	4-May	10:43–11:14	398.8	128.91 ± 44.64
*summer fully-leafed*	clear	0	5-Jul	11:50–12:12	1694.9	23.21 ± 38.68
	partly cloudy	3.5	8-Jun	13:21–13:47	1269	20.35 ± 15.38
	overcast	8	26-Jul	11:49–12:14	186.8	6.72 ± 2.50
*autumnal fully-leafed*	clear	<1	18-Aug	11:12–11:37	1402.3	31.20 ± 38.20
	partly cloudy	3	4-Sep	14:57–15:26	1392.2	21.95 ± 19.83
	overcast	8	21-Aug	12:28–12:55	729	20.03 ± 6.59
*autumnal partially- leafed*	clear	0	26-Oct	11:53–12:28	1140.4	75.17 ± 65.54
	partly cloudy	4	11-Nov	13:15–13:40	281.7	80.45 ± 55.16
	overcast	8	7-Nov	*09:20–09:49	139.4	51.39 ± 24.87

Days chosen to represent clear, partly cloudy, and overcast conditions for each phenoseason.

For values with a ‘*’, no readings were available within ~1hr of solar noon.

**Fig 6:**
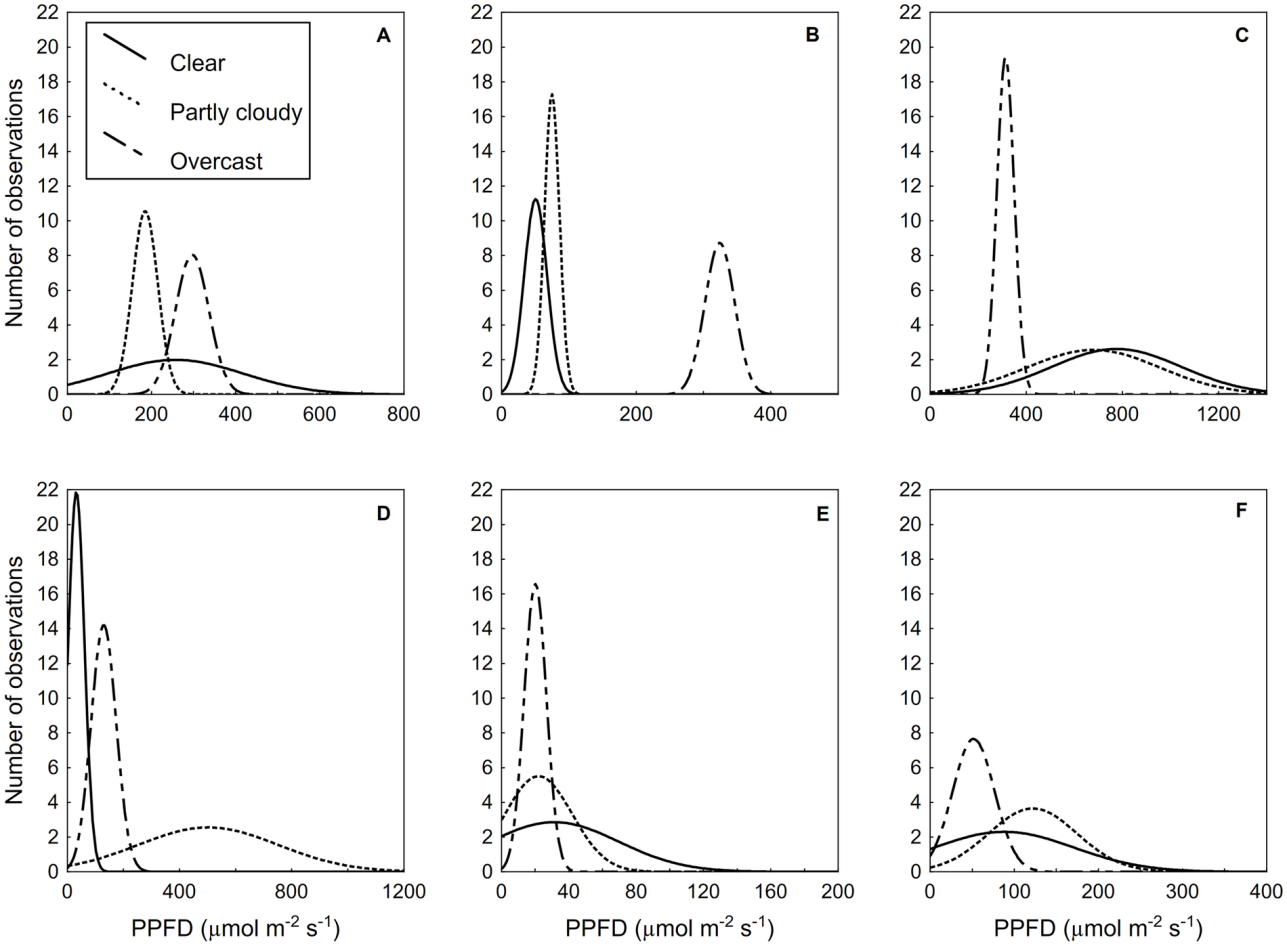
Actual frequency distributions of subcanopy PPFD. Frequency distributions of 25 observations for selected days representing clear, partly cloudy, and overcast sky conditions during the (A) winter leafless phenoseason, (B) spring leafless phenoseason, (C) spring leafing phenoseason, (D) summer leafing phenoseason, (E) the autumnal fully-leafed phenoseason, and (F) autumnal partially-leafed phenoseason. It is critical to note the differences of scale on the *x*-axis for each panel.

The entire winter leafless phenoseason was characterized by low open PPFD values and high mean, median, and minimum subcanopy values (Table 1). During the winter leafless phenoseason, the highest individual measurement of PPFD and the greatest range of PPFD occurred on the selected clear day, whereas greater overall flux and the smallest range of values were measured on the overcast day (Fig 6A). Overcast conditions *increased* radiative energy in the subcanopy relative to a clear or partly cloudy day during the winter leafless phenoseason (Table 2).

The spring leafless phenoseason was brief and cloudy. The lowest open mean and maximum PPFD values and the second highest subcanopy PPFD values were recorded during this phenoseason (Table 1). A likely combination of time, earth-sun geometry, and diffusion of light on the selected overcast day increased overall energy in the subcanopy (Fig 6B). All subcanopy measurements for clear and partly cloudy sky conditions during the spring leafless phenoseason were below 121 μmol m^-2^ s^-1^, while overcast measurements were all above 280 μmol m^-2^ s^-1^ (Table 2).

The highest PPFD values for all subcanopy locations were measured during the spring leafing phenoseason, excluding subcanopy sunflecks and hot spots (Table 1). Subcanopy PPFD values increased for all locations by nearly double during this spring leafing phenoseason, to a median of 341.7 μmol m^-2^ s^-1^ (Table 1). The range of subcanopy PPFD values increased for clear and partly cloudy conditions, while the range of PPFD values for the overcast condition was diminished (Fig 6C). The highest maximum subcanopy values of all the phenoseasons occurred during this phenoseason’s clear and partly cloudy sky conditions, while the overcast condition began to demonstrate lower overall PPFD than the clear or partly cloudy conditions (Table 2).

The number of PPFD measurements classified as hot spots increased by more than one order of magnitude and maximum PPFD remained relatively high for the summer leafing phenoseason (Fig 4). There was a dramatic increase in LAI during the summer leafing phenoseason (see also Fig 3), which in combination with the inherent variability of the partly cloudy sky condition, lead to the overall platykurtosis of the partly cloudy distribution and the continued decrease in flux for the overcast and clear distributions (Fig 6D). For all sky conditions, lower values of PPFD occurred as the leaves matured (Fig 3). Higher maximum values of PPFD occurred in early May, on the partly cloudy and overcast days, than occurred for the clear day in late May (Table 3), likely due to the completion of canopy closure as the month progressed (Fig 6D).

**Table 3:** Summary characteristics of plants for each experiment. ‘SC1’ is the initial stomatal conductance and ‘SC2’ is the secondary stomatal conductance.

Plant ID	# of nodes	Avg. internodal distance (cm)	Cumulative node height (cm)	Avg. SC1 (mmol m^-2^ s^-1^)	Avg. SC2 (mmol m^-2^ s^-1^)	Difference between SC1 and SC2	Root weight (g)	Shoot weight (g)	Root/Shoot Ratio	Total weight (g)	Root dry weight (g)	Shoot dry weight (g)	Root/Shoot dry weight ratio	Total dry weight (g)
	***Experiment 1 (350 μmol m***^***-2***^ ***s***^***-1***^***; 16 hours light*, *16 hours dark)***													
	*Lower Plants (366 μmol m*^*-2*^ *s*^*-1*^*)*													
L2	4	1.9	7.6	169.6	107.5	62.1	1.12	1.31	0.85	2.42	0.18	0.3	0.59	0.48
L3	5	0.94	4.7	174.6	161.9	12.7	0.61	0.48	1.28	1.09	0.06	0.07	0.9	0.13
L4	5	2	10	128.18	42.75	85.43	1.58	1.69	0.94	3.27	0.29	0.34	0.88	0.63
L5	6	2.35	14.1	-	-	-	0.93	0.76	1.22	1.7	0.14	0.16	0.83	0.3
L6	5	1.57	9.4	192.58	58.83	133.75	1.91	1.77	1.08	3.68	0.3	0.41	0.74	0.71
L7	1	1.1	2.2	68.35	32.8	35.55	0.79	0.9	0.88	1.69	0.15	0.16	0.91	0.31
L8	2	0.63	1.9	125.72	31.22	94.5	1.69	2	0.85	3.7	0.38	0.5	0.77	0.88
*Avg*.	*4*	*1*.*5*	*7*.*13*	*143*.*17*	*72*.*5*	*70*.*67*	*1*.*23*	*1*.*27*	*1*.*01*	*2*.*51*	*0*.*21*	*0*.*28*	*0*.*8*	*0*.*49*
	*Elevated Plants (519*.*9 μmol m*^*-2*^ *s*^*-1*^*)*													
U1	4	1.12	5.6	64.05	41.4	22.65	0.22	0.38	0.58	0.6	0.04	0.08	0.54	0.12
U3	3	1.7	6.8	54.27	40.27	14	0.21	0.42	0.5	0.63	0.07	0.11	0.62	0.17
U4	4	1.98	11.9	101.3	63.95	37.35	1.35	0.98	1.37	2.33	0.16	0.23	0.71	0.39
U6	5	1.15	6.9	72.75	54.8	17.95	0.39	0.47	0.84	0.86	0.06	0.1	0.56	0.16
U7	3	0.5	1.5	95.74	69.48	26.26	1.15	0.95	1.21	2.1	0.2	0.26	0.76	0.46
U9	5	2.12	12.7	55.97	51.17	4.8	0.3	0.66	0.46	0.97	0.08	0.17	0.48	0.25
*Avg*.	*4*	*1*.*43*	*7*.*57*	*74*.*01*	*53*.*51*	*20*.*5*	*0*.*6*	*0*.*64*	*0*.*83*	*1*.*25*	*0*.*1*	*0*.*16*	*0*.*61*	*0*.*26*
	***Experiment 2 (150 μmol m***^***-2***^ ***s***^***-1***^***; 12 hours light*, *12 hours dark)***													
	*Lower Plants (118*.*06 μmol m*^*-2*^ *s*^*-1*^*)*													
L1	4	1.75	7	131.43	54.61	76.81	3.52	1.3	2.72	4.81	0.51	0.27	1.92	0.77
L2	1	1	1	189.9	51.93	137.97	1.27	1.22	1.04	2.49	0.19	0.25	0.76	0.44
L3	3	1.17	3.5	152.49	39.19	113.3	1.73	1.31	1.32	3.03	0.3	0.39	0.77	0.68
*Avg*.	*2*.*67*	*1*.*31*	*3*.*83*	*157*.*94*	*48*.*58*	*109*.*36*	*2*.*17*	*1*.*27*	*1*.*69*	*3*.*45*	*0*.*33*	*0*.*3*	*1*.*15*	*0*.*63*
	*Elevated Plants (164*.*5 μmol m*^*-2*^ *s*^*-1*^*)*													
U1	3	1.25	3.75	57.84	41.24	16.6	3.81	2.03	1.88	5.84	0.63	0.56	1.13	1.19
U2	1	2	2	85.81	71.87	13.94	2.43	1.57	1.55	4	0.4	0.41	0.98	0.81
U3	2	1	2	95.11	42.19	52.93	1.18	1.05	1.12	2.23	0.18	0.31	0.59	0.49
U4	3	2.17	6.5	112.19	51.56	60.63	4.11	3.17	1.29	7.28	0.7	0.9	0.77	1.6
Avg.	*2*.*25*	*1*.*6*	*3*.*56*	*87*.*74*	*51*.*71*	*36*.*03*	*2*.*88*	*1*.*95*	*1*.*46*	*4*.*84*	*0*.*48*	*0*.*55*	*0*.*87*	*1*.*02*

With canopy closure during the summer fully-leafed phenoseason, overall subcanopy flux was greatly reduced, and the occurrence of higher-intensity sunflecks became even more apparent in the data. The mean and range of open PPFD values increased during the summer fully-leafed phenoseason, while median subcanopy values were lowest for this phenoseason, at 10.77 μmol m^-2^ s^-1^ (Table 1). Even subcanopy readings of 100 μmol m^-2^ s^-1^ were found to be hot spots during this phenoseason, with the upper quartile being only 18.82 μmol m^-2^ s^-1^ (Table 1). During the summer fully-leafed phenoseason, the highest open and subcanopy maximum PPFD values, and the lowest subcanopy minimum values in the shrub heights were measured (Table 1).

The autumnal fully-leafed phenoseason results were similar to those of the summer fully-leafed phenoseason, except for a decrease in the mean and range of open PPFD due to earth-sun geometry. Mean and minimum subcanopy PPFD values were lowest for this phenoseason due to both the decrease in incoming PPFD and a mature canopy (Table 1). There were fewer hot spot classifications than for the summer fully-leafed phenoseason. Overall subcanopy flux was similarly low during the autumnal fully-leafed phenoseason, so clear and partly cloudy day distributions are right-skewed due to the occasional presence of high-energy sunflecks during these sky conditions (Fig 6E). PPFD values were greatly reduced for all sky conditions but, in contrast to leafless conditions, especially reduced by overcast conditions (Table 2).

The mean and range of open PPFD values continued to decrease to the lowest measured values during the autumnal partially-leafed phenoseason, while means of subcanopy PPFD increased slightly from the autumnal fully-leafed phenoseason, as leaves abscised, but remained low (Table 1). By this point in the season (see dates, Table 2), some other understory plants were still green, but *L*. *benzoin* within the plot had lost 80% or more of leaves. Similar to the autumnal fully-leafed phenoseason, the clear and partly cloudy day subcanopy maximum values were higher and more positively skewed than the fully overcast day due to hot spots in the subcanopy (Fig 6F). Ranges of subcanopy PPFD values for all sky conditions began to increase with leaf abscission in the autumnal partially-leafed phenoseason before the solar elevation began to decrease.

#### PAR variation in the shrub canopy

At the five locations within the site where *L*. *benzoin* was abundant, 1160 measurements were made both within and below *L*. *benzoin* canopy (heights 0 m and 0.75 m, Table 1). These locations are marked in Fig 1C by locations 5, 8, 14, 15, and 23, The most variation between the three heights at these locations occurred during the spring leafing phenoseason. Percentage-of-open PPFD means varied within 6% between the three heights throughout the year (Fig 5B–5D). Results of an ANOVA suggested all but 24% of the variance between heights was explained by phenoseason (Wilks Λ = .24253, F (18, 303.13) = 10.948, *p* = 0.0000). This result was significant at the α = .05 level. When locations were ranked by mean annual PPFD, the five locations with abundant growth of *L*. *benzoin* measured in the 64^th^ percentile of locations receiving the most solar radiation within the plot, *i*.*e*. locations receiving greater than 128 μmol m^-2^ s^-1^. When ranked by median values, four of the five locations fall within the 75th percentile. Similarly, these locations also received greater all-season cumulative flux.

### *Lindera benzoin* treatments in the growth chamber

Visually, the plants from Experiment 2 performed better and achieved greater fitness than the plants from Experiment 1. Mean total weight of plants from Experiment 1 was nearly half that of Experiment 2 (Table 3). Plants from Experiment 1 grew taller, with more nodes, but grew fewer leaves and did not grow as broad as those from Experiment 2.

A Tukey-Kramer Honest Significant Difference test of non-collinear variables found the means of the root-shoot ratios and initial stomatal conductance to be significantly different (α = 0.05) for upper (1U) and lower (1L) plants of Experiment 1. Means of initial stomatal conductance for the upper (2U) and lower (2L) plants of Experiment 2 were significantly different. There was a significant difference between the root-to-shoot ratios for plants grown from 1U (highest) treatment set and plants from 2L (lowest) treatment set (*p* = 0.0130). Total dry weight of both 1U (*p* = 0.0007) and 1L (*p* = 0.0082) treatments were found to be significantly different from 2U. Plants from 2U grew to significantly higher biomass than either treatment from Experiment 1. Finally, significant differences in initial stomatal conductance were found between 1U and 1L and 2U and 2L. (2L-1U, *p* = 0.0022; 2L-2U, *p* = 0.0121; 1L-1U, *p* = 0.0021; 1L-2U, *p* = 0.0177). In other words, there was a significant difference between the stomatal conductivity of plants grown in the 350 μmol m^-2^ s^-1^ light environment and the plants grown in the 150 μmol m^-2^ s^-1^ light environment.

## Concluding discussion

The goal of this study was to better understand how PAR varies within and among phenoseasons in a mid-Atlantic deciduous forest and how this PAR variability might affect woody understory shrubs. Past research has shown that full sunlight may reach to the forest floor for only a second or for several hours, may be direct or diffuse, and may be affected by shadows and by leaf absorption or radiation [6]. Using data collected from 4,592 distinct field measurements from one full calendar year, we found that PPFD values during unfoliated seasons were almost 10 times higher than foliated season PPFD values, except for sunflecks. Our results indicate that small, discrete sunflecks and hot spots during clear days of the summer and autumnal fully-leafed phenoseasons are typically the highest recorded light-energy values, sometimes three orders of magnitude higher than the subcanopy mean PPFD during the foliated seasons. These large changes in PPFD can occur over distances as small as a centimeter.

Biologically, an understory species must make the most of the peak in annual energy which occurs because of the combination of an increase in insolation and an unfoliated canopy, as this is the time for germination and reproduction, activities which require the most energy. In a natural setting, *L*. *benzoin* has a distinct advantage by blooming early. Within the study plot, *L*. *benzoin* began to bloom in the first week of April, while canopy trees did not begin bud burst until the end of April. Leaf-out of *L*. *benzoin* occurs early in the spring leafing phenoseason, weeks before leaf-out of trees in the canopy, and just before the subcanopy radiation begins to significantly decrease. Other understory plants in the plot begin to bloom later in the season, some reaching full bloom as late as mid-May. *L*, *benzoin* make use of the high amounts of PAR reaching the forest floor during the spring leafless and early spring leafing phenophases. This early bloom is also ecologically beneficial for early pollinators, who may have access to only limited resources so early in the season.

**Fig 7:**
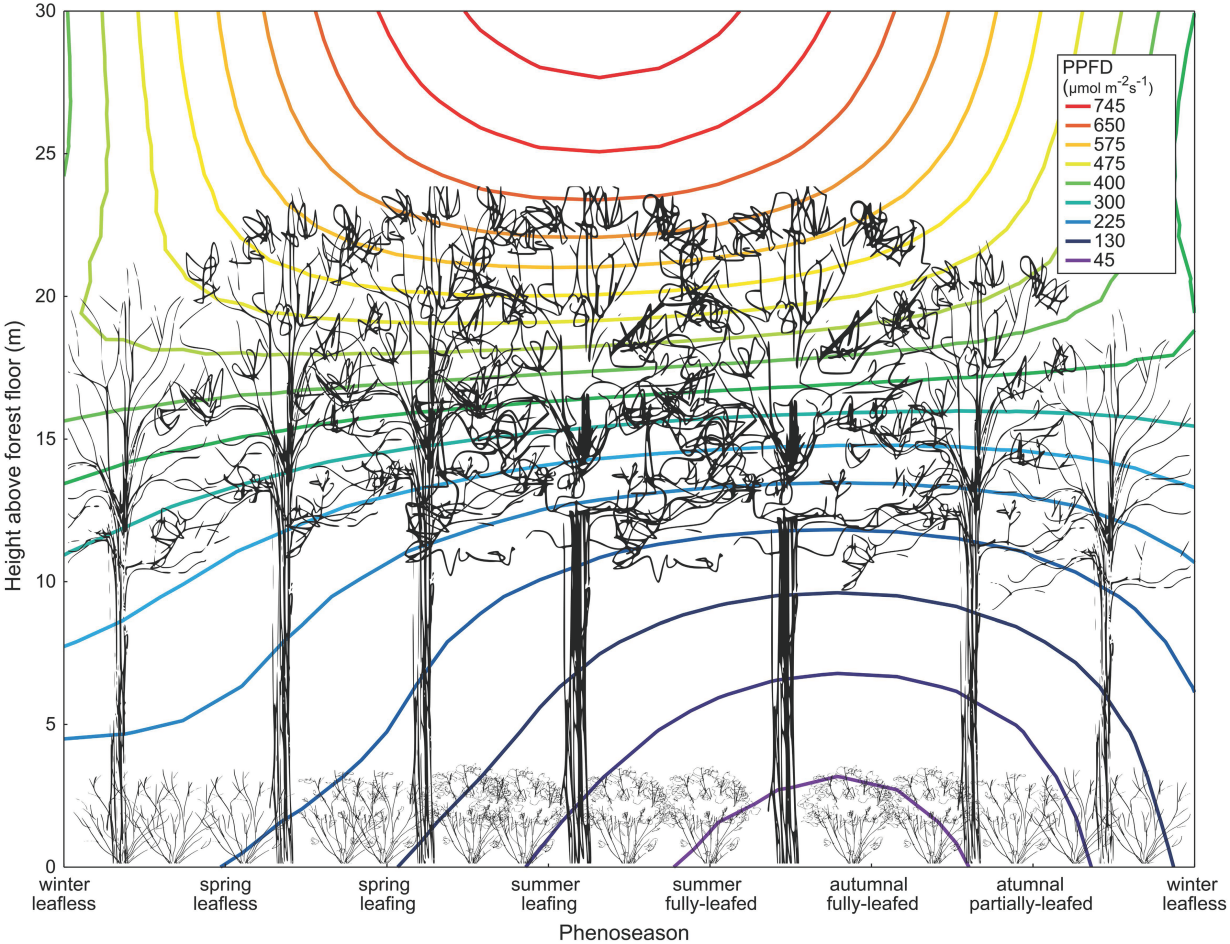
Annual change in average daily PPFD below the forest canopy. Subcanopy plants (not to scale) receive peak available energy during the spring leafless and leafing phenoseasons, energy they must use for critical physiological functions such as germination and reproduction.

Inspired by the work of Hutchison and Matt [2], Fig 7 uses our own interpolated field data to illustrate this important phenological occurrence using contours of measured incoming PPFD throughout the year at a given height above the forest floor. Changing contours above the canopy are indicative of changes in earth-sun geometry, while changes beneath the canopy are, as we have already demonstrated, widely controlled by phenoseason *i*.*e*. the combination of canopy state and earth-sun geometry. Mean subcanopy values of PPFD are low year-round, and decrease to less than 75 μmol m^-2^ s^-1^ during the summer and autumnal fully-leafed phenoseasons. It is during the crucial peak in solar energy, occurring between the spring leafless and spring leafing phenoseasons, that *L*. *benzoin* receives the necessary light energy to complete its resource-intensive growth and reproductive functions.

Fig 7 also illustrates another interesting finding regarding the amount of light intercepted by woody areas in leafless phenoseasons. Clearly, subcanopy PAR remains low year-round but our results quantitatively indicate that less than 50% of incident PAR ever makes it to the forest floor, even during leafless phenoseasons. This means that up to 50% of the incident PAR is intercepted by woody frames of trees and shrubs in our field site. Without question, woody area index must be considered in future models of light attenuation through forest canopies, although calculation of such a complex variable and its contribution has yet to be fully integrated [32].

Complementing the field measurements, the purpose of the growth chamber treatments was to determine how changes in available light energy in the growth environment affect overall plant health. What we have shown is that in the short-term these shade-adapted plants significantly modify their physical regulation for survival in high light environments. We have also shown that over time, *Lindera benzoin* in low-light conditions had significantly higher biomass than those in high-light conditions. These results suggest that, as could only be inferred from our field site, the ecological niche for these plants is very specific in terms of light intensity. We expected that spectral composition and quantity would not only affect the growth of *L*. *benzoin*, but also its health and ability to thrive. In addition to causing physiological and chemical adaptations in plants, light quality can also influence the expression of morphological genes [33] and the production of immunological plant hormones such as salicylic acid and jasmonic acid [34,35]. Luken *et al*. [25] showed that *L*. *benzoin* grown in shade houses demonstrated greater amounts of new stem growth, lower stomatal density, highest photosynthetic rate and lower leaf thickness at light levels between 191 and 391 μmol m^-2^ s^-1^ than plants grown in light levels above or below these thresholds In this case, experimental plants showing the most physical success in terms of biomass received 164.5 μmol m^-2^ s^-1^ for 12 hours (2U). Although this value is lower than what might be considered peak efficiency for this type of plant, it was a closer-to-natural scenario (~ 130 μmol m^-2^ s^-1^, in our field site) than the other photoperiod-photointensity combinations. Though the lower set from Experiment 1 experienced photointensity closer to the value of that found to be conducive for peak efficiency (366 μmol m^-2^ s^-1^), understory plants do not likely experience such an intense photon flux for more than a few hours, and especially not for 16 hours straight, every day for weeks. As Chazdon and Pearcy [6] suggest, it is possible many plants may experience greater success in the field in terms of carbon gain because of precise temporal light sequences, rather than cumulative light exposure alone. That being the case, a growth chamber’s rapid increase from darkness to a controlled photointensity that remains constant for a full 12 or 16 hours before instantly returning to complete darkness is hardly representative of the highly variable temporal light sequences we now know to be occurring in the field.

We demonstrated that phenoseason (*i*.*e*., the combination of canopy state and celestial geometry) may account for nearly 75% of the variation between canopy heights in this mid-Atlantic deciduous forest. The influence of phenoseason on the differences of PAR received at three heights within the subcanopy, and the critical nature of its combined inputs to subcanopy plants highlights the importance of this construct to study the physiological ecology of understory plants. The other 26% may be variation caused by sky conditions, slope, aspect, and canopy species distribution. While understory PPFD is typically between 20 and 40% of open PPFD, values of PPFD in the shrub canopy are often only 5–12% of the incident PPFD. Additionally, our results agree with Hutchison and Matt’s [2] findings that winter leafless overcast conditions increased subcanopy radiation, whereas summer overcast conditions decreased subcanopy radiation. We hypothesized that the re-distribution of PAR by canopy species would affect the growth and distribution of the shrub *L*. *benzoin* in the subcanopy by restricting a primary growth-limiting resource. Although no direct conclusions could be made about species dependencies (*i*.*e*. disparities in subcanopy light availability under *L*. *tulipifera* vs. *F*. *grandifolia*), the fact that all five subcanopy locations with greater occurrence of *L*. *benzoin* receive in the upper 36% of annual PPFD suggests that variance in the crown shape and LAI of canopy species above does impact the growth of shade-adapted species in the understory.

Ecological climatology involves the bidirectional coupling of ecosystems and climate, highlighting the interconnections among populations at all scales, from individual, to stand, to regional, to global [19]. In these two complementary experiments, we have demonstrated these critical linkages. We have shown how inherently variable subcanopy light is; a change in any one of the biotic or abiotic factors controlling phenoseason could result in large changes to the light regime below the canopy. We have also shown how biophysically sensitive an individual plant species, such as *L*. *benzoin* within our plot, can be to light regime changes as seemingly small as 50 μmol m^-2^ s^-1^. On a large scale, the photosynthesis of these canopy and subcanopy plants plays a large role in the carbon and water cycles, the maintenance of which are of global importance [19,36,37]. It is important to continue to better our understanding of the nuances of such processes, how these processes may change in the future, and how future changes to these processes may affect species composition, plant structure, function, production, and even periodicity of phenophases of a forest stand, to better understand the larger role plants play in the future of the Earth’s climate.
